# Iatrogenic colorectal Kaposi sarcoma complicating a refractory ulcerative colitis in a human immunodeficiency negative-virus patient

**DOI:** 10.11604/pamj.2013.15.154.2988

**Published:** 2013-08-29

**Authors:** Lamine Hamzaoui, Houda Kilani, Mahdi Bouassida, Moufida Mahmoudi, Emna Chalbi, Karima Siai, Heykel Ezzine, Hassen Touinsi, Mohamed M'Saddak Azzouz, Sadok Sassi

**Affiliations:** 1 Gastroenterology department, Mohamed Tahar Maamouri Hospital, Nabeul, Tunisia; 2 Histopathology department, Mohamed Tahar Maamouri Hospital, Nabeul, Tunisia; 3 General surgery department, Mohamed Tahar Maamouri Hospital, Nabeul, Tunisia

**Keywords:** Kaposi's Sarcoma, ulcerative colitis, Human Herpes Virus-8, Infliximab, immunosuppression

## Abstract

Kaposi sarcoma is a mesenchymal tumor associated to a human herpes virus-8. It often occurs in human immunodeficiency virus-positive subjects. Colorectal localization is rare. We report the case of a colorectal Kaposi sarcoma complicating a refractory ulcerative colitis treated with surgery after the failure of immunomodulator therapy in a human immunodeficiency virus-negative heterosexual man.

## Introduction

Kaposi's sarcoma (KS) is a mesenchymal tumor, arising predominantly in the skin but which can affect any organ system. It's associated to human herpes virus-8 (HHV8) [1, 2]. Patients with inflammatory bowel disease, in particular ulcerative colitis (UC), are often treated with immunosuppressive therapy and can develop colorectal KS [3]. We report the case of a human immunodeficiency negative-virus (HIV) man, with a severe refractory UC, who was treated with steroids, azathioprine and infliximab (IFX). Failure of medical treatment indicated surgery. Histological examination of the colon revealed KS.

## Patient and observation

**Figure 1:**
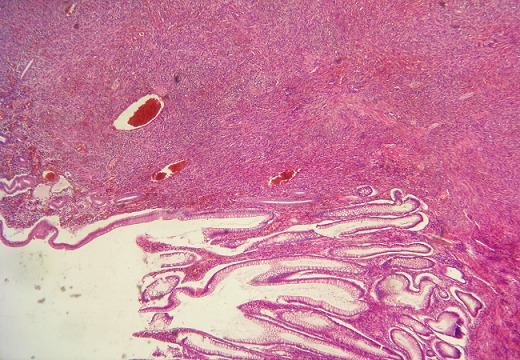
Histology (H and E stain x 40): Spindle cell proliferation with vascular channels infiltrating a colonic mucosa

**Figure 2:**
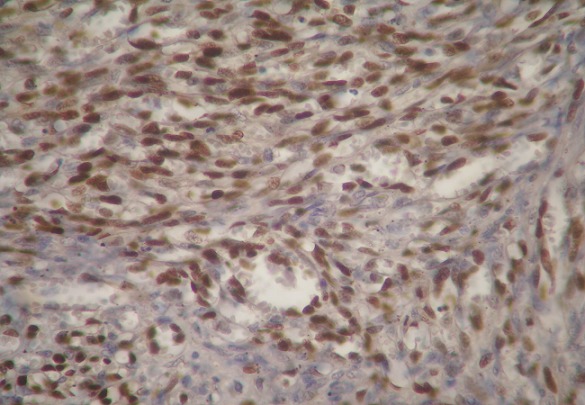
HHV8 immunohistochemical stain: strong nuclear positivity in spindle tumor cells x200

**Figure 3:**
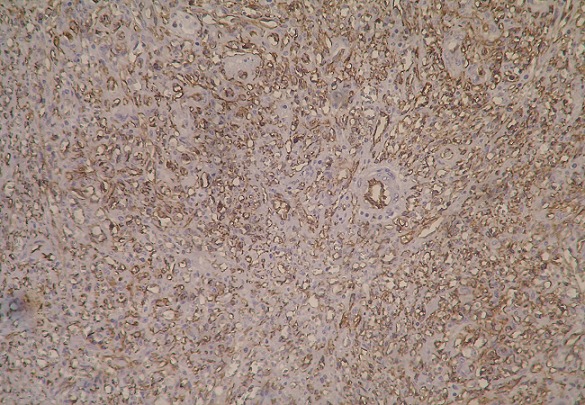
Fig CD31 immunohistochemical stain: positivity in vascular channels x40

A 30-year-old heterosexual man, without familial and personal history, was diagnosed with rectosigmoid ulcerative colitis since 2010 revealed by chronic bloody diarrhea with abdominal pain and weight loss. He had a 3 pack-year history of smoking (stopped 3 years ago). He was initially treated with oral and local mesalamine with a good response. In 2011, he was treated 3 times with steroids (1mg/kg/day of prednisone) for a moderately active disease without a complete relief of diarrhea. In December 2011, at physical exam, his body mass index was 17kg/m^2^. Abdominal exam was normal. There was no skin lesion. Iron deficiency anemia (11g/dL) and signs of inflammation: erythrocyte sedimentation rate (ESR) of 37mm and C - reactive protein (CRP) of 74mg/L were noted. Albumin level was 25g/L. Ileocolonoscopy showed large superficial ulcerations, spontaneous bleeding and pseudo-polyps. Lesions were mainly located in the rectosigmoid colon and the colitis was extended to the hepatic flexure. Histology found clear signs of active UC with no signs of malignancy. Oral corticosteroids were prescribed with a good initial response. At week 4 of corticosteroid therapy, we noted a relapse of bloody diarrhea, inflammation and aggravation of anemia (7,3g/dL). A left-sided colonoscopy didn't show severe endoscopic signs. Copro-parasitological examinations were negative. Cytomegalovirus testing was negative too. Detection of *Clostridium difficile* toxins was not done. Intravenous corticosteroids and parenteral nutrition were prescribed during 1week. At week 5 of corticosteroid therapy, another relapse occurred (7 to 8 stools daily, anemia 6g/dL, ESR 40mm, albumin 17g/L) and we had considered that it was a refractory severe UC. As the patient was reticent to surgery, medical treatment with immunomodulators was indicated. Assessment before immunosuppressive therapy was normal. He received blood transfusion, albumin infusion, parenteral nutrition and intravenous corticosteroids (with progressive decreasing doses). After improvement in health status, anemia (9g/dL) and albumin concentration (37g/L), azathioprine (2,5mg/kg/day, total of 3 months of treatment) and IFX (induction regimen with 5mg/kg, weeks 0, 2 and 6) were prescribed with unchanged disease activity. Drug failure led to a surgical treatment. A subtotal colectomy with double stomy of the ileum and of the sigmoid colon was performed. Colon macroscopic examination revealed multiple polypoid red lesions associated with large ulcerations. Histologic examination of polypoid lesions showed fusocellular spindle cells proliferation with a slit like vascular channels and extravased red blood cells consistent with the diagnosis of KS, associated with typical features of UC (Figure 1). Immunolabelling for HHV8 stained the nuclei of the spindle cells (Figure 2, Figure 3). Involvement in 1 of the 25 perivisceral lymph nodes isolated was demonstrated. The patient underwent mucosal proctectomy and ileoanal anastomosis.

## Discussion

There are four clinical variants of KS with distinct clinical and epidemiological characteristics: classic, endemic, acquired immunodeficiency syndrome and iatrogenic [4]. Iatrogenic KS has been described in kidney and liver transplant recipients and also auto-immune diseases [5, 6]. KS of gastrointestinal tract is rarely seen in the developed countries. Involvement by KS has been noted in 80% of patients with visceral disease in endemic areas [7]. Case studies have reported colorectal KS associated with inflammatory bowel disease in HIV-negative patients (since until now 11 observations). Most of cases were refractory UC on immunosuppressive therapy [8]. Our patient has received a triple immunosuppression (steroids, azathioprine and infliximab). It's the second case occurring into IFX. The diagnosis is difficult to establish in the absence of skin lesions, as in our patient. Nodular and polypoid lesions are observed in endoscopy and biopsies may fail to sample diagnostic tissue before tumor infiltration of the mucosa [8]. Intraluminal polyps are red or blue, due to high vascular and conjunctive tissue proliferation. Distinction from pseudo-polyps is difficult [9]. Seven months before diagnosis, the colonoscopy showed polypoid lesions, considered as inflammatory pseudo-polyps by the histologist. Immunohistochemistry of HHV-8 is helpful in the histological assessment of the lesions and may demonstrate infiltration of the mucosa by KS [8]. HHV-8 was positive in our case. Proctocolectomy associated to immunosuppressor withdrawal is usually effective to treat both UC and KS. After subtotal colectomy and confirmation of KS associated to UC, the patient will undergo proctectomy.

## Conclusion

Although it is rare, it is important to consider a concomitant KS in patients with refractory severe ulcerative colitis, on immunosuppressor therapy, independently of HIV status.
